# High Prevalence of Iron Deficiency among Educated Hospital Employees in Switzerland

**Published:** 2011-06

**Authors:** Reto A. Schuepbach, Lukas Bestmann, Markus Béchir, Jörg Fehr, Esther B. Bachli

**Affiliations:** 1 *Medical Clinic, Department of Medicine, University Hospital Zurich, Zurich, Switzerland;*; 2 *Institute of Clinical Chemistry, University Hospital Zurich, Zurich, Switzerland;*; 3 *Division of Surgical Intensive Care, University Hospital Zurich, Zurich, Switzerland;*; 4 *Division of Haematology, Department of Medicine, University Hospital Zurich, Zurich, Switzerland*

**Keywords:** iron deficiency, anaemia, hospital epidemiology

## Abstract

Iron deficiency is known to cause symptoms such as fatigue, depression and restless legs syndrome resulting in impaired quality of life and working capacity. We sought to examine the iron status of reportedly healthy individuals by a framed study design in 58 highly educated Swiss hospital employees and to compare the use of non invasive tests for assessing iron deficiency (ID). A structured interview was used to assess health status, nutritional intake and potential blood loss, blood counts as well as parameters proposed to diagnose iron deficiency were determined. All subjects felt well and were working at their maximum capacity. The male subjects were neither anaemic nor had decreased iron parameters however 50% (23/46) of the women had a serum ferritin of below 22 μg/L, still 33% (15/46) of the women had a ferritin value below the more stringent cut off value of 15 μg/L. In 15% (7/46) of the women we diagnosed iron deficient anaemia. Red meat consumption correlated with ferritin values as did the menstrual blood loss which was estimated by asking the amount of tampons used. Of the additionally analysed iron parameters only the percentage of hypochromic erythrocytes, soluble transferrin receptor and transferrin values were significantly correlated with ferritin and reached an AUC_ROC_ of ≥0.7 indicating good predictive tests. Nevertheless neither soluble transferrin receptor nor transferrin showed diagnostic advantages for the diagnosis of ID compared to ferritin alone or together with erythrocyte parameters. Working in a hospital environment and having access to health education does not seem to correlate with prevention of ID or ID anaemia in female hospital employees.

## INTRODUCTION

One quarter of the world’s population is thought to be iron deficient (1, 2). Highest prevalence is reported from developing countries such as Asia and Africa and iron deficiency (ID) has been associated with poor education (3). In Europe the prevalence of ID has been reported to be as high as 12 to 40% (4, 5). In the United States, ID has a prevalence of 1-4% in males, 9-11% in menstruating and 5-7% in postmenopausal women (6); 2 to 4% of these women have iron deficiency anaemia (IDA). Increasingly, ID is recognised to cause symptoms such as fatigue, depression and restless leg syndrome (7-10), symptoms that result in impaired quality of life and working capacity.

The gold standard for determination of depleted iron stores is the lack of stainable iron in the bone marrow (11-14). However, this is an invasive and costly examination. Less invasive laboratory tests such as determination of serum iron, transferrin, transferrin saturation, ferritin, soluble transferrin receptor, soluble transferrin receptor index and reticulocyte parameters are available and are proposed as useful in detection of iron depletion before the onset of anaemia. However, except for ferritin (15-18) these tests are considered too expensive and time consuming and have not all been validated against stainable iron in the bone marrow (5, 19-22). Furthermore, comorbidities such as inflammatory or liver diseases may influence these parameters (11, 22). In healthy subjects serum ferritin has been shown to have the highest correlation with iron stores (23, 27). The only two conditions other than iron deficiency known to lower serum ferritin (28) are hypothyroidism and ascorbate deficiency which have very rarely confounded the interpretation of ferritin values (29). Currently ferritin is the preferred non invasive parameter used to diagnose ID in healthy subjects. However the wide range of ferritin cut off values proposed makes estimation of ID still challenging. The reason for the wide range of cut off values of ferritin are i) the fact that ferritin can be induced under chronic conditions (30), and the fact that ID develops over 3 stages. First only the iron storage compartment is affected (ID stage I). Then the compartment for iron transportation becomes affected and as a sign of iron deficient erythropoiesis (IDE) hypochromic and microcytic erythrocytes are found (ID stage II). Finally, shortage within the functional compartment results in IDA (stage III). Initial evaluation of ferritin cut of values followed the concepts of conventional reference values and established distinction of non anaemic subjects and patients with IDE (ID stage II) or IDA (ID stage III) (11, 15, 31). More recent studies established higher cut off values, allowing the diagnosis of patients with ID stage I (5, 14).

We sought to examine the influence of health care education on iron status and to evaluate the current non invasive tests for diagnosing ID in healthy hospital employees.

## METHODS

### Subjects

This framed study relied on a population of 60 hospital employees that were initially recruited to validate a clotting device. The volunteers consisted of nurses and doctors. All declared to be in perfect health and good general physical condition. They signed a written informed consent to allow venipuncture and completed an anonymous standardised questionnaire. We evaluated eating habits, possible blood loss such as menstrual or intestinal blood loss, pregnancy or former pregnancies, accidents, blood donation or blood transfusions as well as surgery. Additional information was obtained concerning any co-morbidities, regular medication and exercise.

### Laboratory methods and sample analysis

Venous blood was drawn into EDTA, heparin and plain vacutainer tubes (Becton-Dickinson, San Jose, CA, USA) and all analyses were carried out within 6 hours of blood collection. Volunteers were screened for signs of inflammation (C-reactive protein and leukocyte count) and haemolysis (haptoglobin and reticulocyte count). Haemoglobin (Hb), red cell indices such as mean corpuscular volume (MCV), percentage of microcytic and hypochromic erythrocytes, reticulocyte count, reticulocyte haemoglobin content (CHr) and leukocyte count were determined by an Advia 120 haematology analyser (Bayer Diagnostics, Germany) according to the manufacturers instruction.

Serum ferritin and C-reactive protein were measured by an Access Immunoanalyser (Opera, respectively Advia Centauer; Bayer Diagnostics, Germany). Serum iron (Fe) was analysed by a commercially available fluorescence assay on a Modular System P (Roche Diagnostics, Mannheim, Germany). Serum transferrin (sTf) and soluble transferrin receptor (sTfR) were measured immunoturbidimetrically on Roche’s E170 Modular and on the Integra 800 respectively (Roche Diagnostics, Rotkreuz, Switzerland and Mannheim, Germany).

Transferrin saturation (SsTf) was calculated as follows:

SsTf=Feμmol/L×0.5sTf μmol/L

The soluble transferrin receptor index (sTfRI) was calculated as proposed by Punnonen *et al*. (32):

sTfRI=sTfRμg/Llog Ferritin μg/L×1000

### Evaluation of nutritional habits and iron loss

To assess iron intake, the volunteers were asked to provide the estimated amount of red meat in grams consumed per week as well as information pertaining to consummation dairy products, blood transfusions and pharmacological iron substitution. Iron loss was evaluated by asking the menstruating female volunteers for the number of tampons used per month and information regarding pregnancies. In addition we asked for information about intestinal blood loss, intestinal pathologies, and recent surgery or blood donation. For anamnestic variables with significant correlation to ferritin a multiple regression model was fitted in order to best predict ferritin values out of anamnestic information.

### Statistical analysis

Data was analysed using the SPSS (SPSS 11.5 Inc., Chicago, Illinois, USA) and the NCSS (Kaysville, Utah, USA) software packages. To compare serum ferritin to other iron parameters Spearman’s correlation coefficient and receiver operator curves (ROC) with areas under the curve (AUC_ROC_) were calculated. Significance was either determined by probability calculation (always 2-sided tests) or estimation of the 95% confidence intervals. Fitting of multiple regression models the NCSS software was used.

## RESULTS

### Characteristic of the study subjects

We sought to determine the extent of iron deficiency among hospital employees. Using a framed study design 60 volunteers that had been randomly selected to validate a clotting device were enrolled into the actual study. Of all enrolled individuals everyone except for one woman with a known asymptomatic β-thalassaemia and one man with elevated CRP and ferritin values were analysed. Three of the individuals that were analysed had following co-morbidities: two women received calcium antagonist therapy because of arterial hypertension and one man had only one kidney but normal kidney function.

Females accounted for 79% (46/58) and men for 21% (12/58) of the volunteers with a combined average age of 34.2 years (SD 7.6, range 24-55). Seven women had given birth within the last 2 to 5 years before participating in this study, however no one was pregnant while participating in the study. None of the volunteers had signs of inflammation (C reactive protein >5 mg/L, leukocyte >11000/mm^3^) or haemolysis (haptoglobin <0.5 mg/L and/or reticulocyte counts > 80’000/μL). The study population’s characteristics are summarized in Table 1.

**Table 1:** Baseline characteristics of study subjects, (mean ± SD)

Variable	Men (*n*=12)	Women (*n*=46)	*p* value^a^

Age (year)	34.1 ± 5.9	34.2 ± 8.0	0.95
Hemoglobin (g/dl)	14.9 ± 0.8	12.8 ± 1.3	<0.001
MCV (fL)	85.7 ± 1.7	87.2 ± 6.0	0.43
Ferritin (μg/L)	86.8 ± 27.5	26.4 ± 20.0	<0.001
Iron (μmol/L)	18.7 ± 3.6	16.6 ± 6.6	0.29
Transferrin sat. (%)	32.2 ± 8.0	25.6 ± 10.8	0.05
sTfR (mg/L)	3.3 ± 0.7	3.8 ± 2.1	0.44

a *p* values are tested for two sided t test of mean differences between gender.

### Serum ferritin

**Figure 1:**
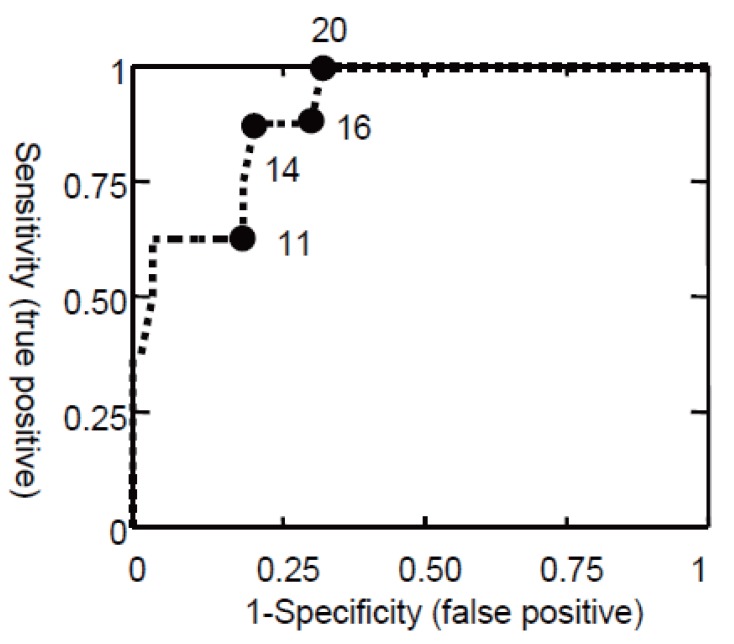
ROC curve for serum ferritin in diagnosing IDE and/or IDA (ID stage II or higher). Cut off values for ferritin are given in μg/L. The estimated AUC is 0.85 (95% CI 0.62 to 0.99; n=58).

Ferritin cut off values between 12 μg/L to 70 μg/L have previously been proposed to be diagnostic for depleted iron stores (11, 16, 17, 32, 33); we used the commonly used cut off value of 22 μg/L, which has been proposed to distinguish in otherwise healthy individuals between subjects without iron depletion and patients with ID stage I or higher (5, 34). We found that none of the study subjects with a ferritin value above 22 μg/L was anaemic or had signs of iron deficient erythropoiesis (absence of ID stage II or higher). None of the men had a ferritin value of below 22 μg/L but 50% (23/46) of the females had a ferritin value below this cut off. Of these 7 were anaemic and one presented with microcytosis. Lowering the cut off point to a more sensitive value of 15 μg/L resulted in 2 false negative tests, wrongly excluding one woman with microcytic anaemia (ID stage III) and one with microcytosis (ID stage II). This finding is consistent with reports from otherwise healthy individuals that a quarter of the female population has signs of iron deficient erythropoiesis (ID stage II or higher) and absent stainable bone marrow iron (15) despite having serum ferritin levels above 15 μg/L. To better visualize the influence of the cut off point on the sensitivity we calculated the receiver operator curves ROC as provided in Fig. 1. The overall accuracy of a test can be described as the area under the ROC curve (AUC); the larger the area the better the test (35). In our test population the AUC for ferritin to ID stage II or higher was found to be 0.85 (95% confidence interval 0.62 to 0.99).

Given the high prevalence of ID, IDE and IDA we considered our study population suited to perform comparative analysis between tests proposed to diagnose ID.

### Correlation between ferritin, serum iron, transferrin, transferrin saturation, soluble transferrin receptor and reticulocyte parameters

**Figure 2:**
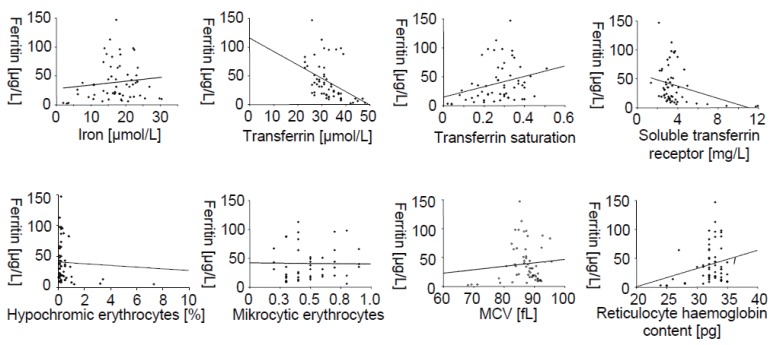
Correlation between ferritin and alternative tests; iron, transferrin, transferrin saturation, soluble transferrin receptor, percentage of hypochromic erythrocytes, microcytic erythrocytes, mean corpuscular erythrocyte volume (MCV) and reticulocyte hemoglobin content. Parameter specific values for correlation, the 95% CI for the slope and R-squared are given in Table 2; n=58.

To compare the tests proposed to reflect iron stores, correlations between ferritin and alternative tests such as serum iron, transferrin, transferrin saturation, soluble transferrin receptor and erythrocyte and reticulocyte parameters were calculated (Fig. 2). Values for correlation, the 95% confidence interval for the slope and R-squared are summarized in Table 2. Using linear correlation we estimated how well the value of an alternative test can be predicted by knowing the value for ferritin. We found that only correlations to transferrin, transferrin saturation, soluble transferrin receptor and hypochromic and microcytic erythrocytes were significant (*p*<0.05) although weak.

**Table 2:** Correlation between ferritin and alternative tests for diagnosis of ID

Test	Correlation	95% CI^a^	R^2^

Iron	0.123	-0.76 to 2.07	0.015
Transferrin	-0.453	-3.57 to -1.11	0.205
Transferrin saturation	0.291	10.7 to 168	0.084
sTfR	-0.314	-9.65 to -1.02	0.099
Hypochromic erythrocytes	-0.287	-2.60 to -0.15	0.083
Microcytic erythrocytes	-0.269	-3.56 to -0.07	0.072
MCV	0.099	-1.00 to 2.20	0.009
Reticulocyte haemoglobin	0.246	-0.17 to 6.50	0.060

a lower and upper limit of the 95% confidence interval for the slope.

Since correlation tests examine the effect over the whole scale of the parameters, good prediction at one end of the scale might be cancelled out by poor prediction at the other end. Sensitivity/specificity analysis could therefore be more valuable to evaluate alternative tests.

### ROC curves for serum iron, transferrin, transferrin saturation, soluble transferrin receptor and reticulocyte parameters

**Figure 3:**
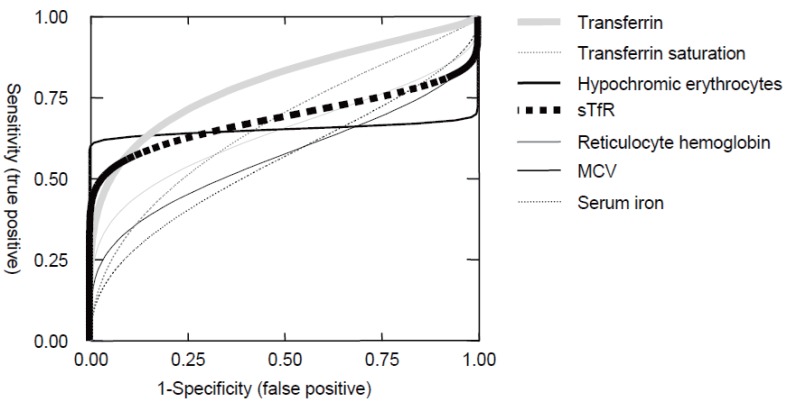
Using a ferritin cut off value of 22 μg/L the study population was split into subjects without ID and patients with ID. Receiver operator curves were calculated for iron, transferrin, transferrin saturation, soluble transferrin receptor, hypochromic erythrocytes, mean corpuscular erythrocyte volume and reticulocyte hemoglobin content. For each test parameter the estimated AUC with 95%CI is summarized in Table 3; n=58.

A serum ferritin level of 22 μg/L was used to split the study population into two groups, iron deficient (ferritin below 22 μg/L) and non iron deficient volunteers. The accuracy of alternatively testing serum iron, transferrin, transferrin saturation, soluble transferrin receptor and reticulocyte parameters was then estimated by calculating the AUC in receiver operator curves (Fig. 3); calculated values are given in Table 3. Only transferrin yielded a reasonable AUC of 0.8. AUCs of transferrin saturation, soluble transferrin receptor and the percentage of hypochromic erythrocytes were around 0.66. The ROC of hypochromic erythrocytes is of particular interest: Despite an AUC of 0.66 only, stringent cut off values are highly specific at coast of a sensitivity of 60% only.

**Table 3:** AUC values for tests proposed to diagnose iron deficiency

Test	AUC	95% CI for AUC

Transferrin	0.796	0.642 to 0.887
Transferrin saturation	0.666	0.549 to 0.758
Hypochromic erythrocytes	0.660	0.483 to 0.785
Soluble transferrin receptor	0.656	0.549 to 0.758
Reticulocyte Haemoglobin	0.643	0.494 to 0.756
MCV	0.569	0.474 to 0.651
Iron	0.559	0.457 to 0.646

### Red meat consumption and blood loss

The average red meat consumption in our study population was 290 g/week, 280 g/week for females and 330 g/week for males and was more than 5 times lower than reported for other Swiss study populations (36). Meat consumption was especially low among the 15 volunteers with ferritin levels below 15 μg/L and was 232 g/week on average. Of these, 3 never ate red meat, 6 consumed below the average and 6 above the average of 290 g/ week. Females with ferritin values above 22 μg/L consumed an average of 232 g/ week but this group still contained six vegetarians. The amount of red meat consumed and ferritin values correlated significantly but weakly (R-squared 0.025).

Women were asked for the number of tampons used per month as a surrogate of menstrual blood loss. Women with ferritin values below 15 μg/L used significantly more tampons than women with values above 15 μg/L (Mann-Whitney Test, *p*=0.031) and the number of tampons used correlated weakly but significantly with ferritin values (R-squared 0.104).

To test if the parameter of ferritin values in women could be better predicted if the data from red meat consumption and the number of tampons used would be combined we performed a multiple regression model where the estimated model

**Figure 4:**
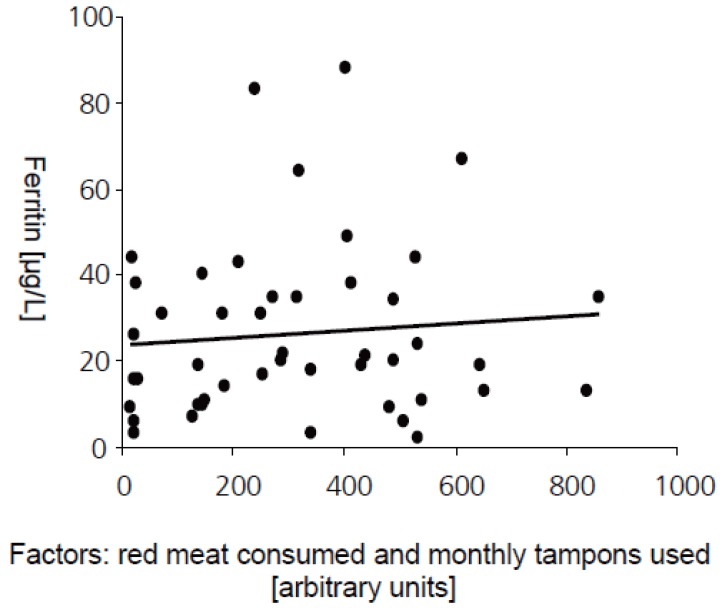
The combined information on red meat consumption and number of monthly used tampons was correlated to the value of serum ferritin in femals (R-squared 0.12; n=46).

Ferritin [μg/L] = 34 + (1.9 × meat/week[g]) – (0.71 × number of tampons used)

had the best fit but still resulted in a weak correlation (R-squared 0.12, Fig. 4). All other items we asked for (pregnancy or former pregnancies, consumption of diary products and blood donation) were not found to significantly contribute to the prediction of ferritin.

## DISCUSSION

We found a prevalence of 50% for ID in a sample of healthy female hospital employees. If a more specific cut off point of 15 instead of 22 μg/L for ferritin was used the prevalence was still as high as 33%. Similarly high prevalence was reported in Canada and Finland and was found to be up to 40% in menstruating women and 3% in men (5, 37). These data contrast to reports from the United States where the prevalence was estimated to be 9-11% in menstruating, 5-7% in postmenopausal women and 1-4% in men (6).

Our study was a survey and not a representative analysis of the prevalence of ID and might therefore suffer from selection bias (with hospital employees suspecting to suffer from ID more readily participating). However the subjects were primarily recruited to standardize a clotting device and since the individuals were explicitly advised to enrol only if they felt to be in perfect health we assume to be reporting data from a very healthy population. In contrast we rather speculate that in a less selected population of hospital employees ID would be even more prevalent.

The consumption of red meat which falls below the average for both, Swiss and European populations might in part explain the high prevalence of ID in our study population. Why health care workers with normally good education in questions concerning nutrition (3, 15) would not sufficiently substitute their diet with iron rich food other than red meat remains unclear. Nevertheless one is tempted to claim nutritional habits as a likely factor, since we and others (4) found a correlation between ferritin and red meat consumption and because the prevalence of ID was reported to be low from countries with high red meat consumption such as the United States (6).

Most textbooks cite a higher prevalence of ID in menstruating women, which seems to be associated with the extent of blood loss and the number of pregnancies. We confirm a significant correlation of blood loss as calculated by tampons used during menstruation and ferritin values. Recently the effect of female specific factors has been evaluated and found to only partially explain the variation of ferritin levels among twins (38). Whitfield *et al*. claim that genetic differences from partially yet unknown genes must contribute. Whether such genetic factors account for our findings remains open since we did not address this issue.

ID has been associated with depression, chronic fatigue, impaired endurance performance and restless leg syndrome, all of which lead to sleep disturbances (7-10). Iron supplementation has been reported to improve such symptoms (9). Surprisingly the volunteers in our study all claimed to be well and capable. One could speculate that our study is biased by only enrolling subjects that still felt well but that may develop depression and similar symptoms over time. Such reflections are especially worrisome in view of the high burn out rates among health care workers (39, 40). Additional research will be required to clarify if ID and burn out among hospital employees are potentially linked.

Estimation of the iron status in an individual remains a difficult task. Invasive bone marrow biopsy is considered the gold standard but has been reported to be inaccurate in more than 30% of the cases (41). In addition poor quality of the biopsy material can result in diagnostic failure in up to 35% of the cases (25). Serum ferritin is widely recognized as a valuable parameter when estimating the iron stores (42), however determining the cut off value discriminating between individuals with normal serum stores from those with ID remains troublesome. Our data confirm previous reports, that functional iron depletion (ID stage II or higher) is absent in individuals (with no chronic inflammation) with serum ferritin values above 22 μg/L (5, 34).

To better evaluate individuals with ferritin values close to a cut off value of 22 μg/L the additional evaluation of the sTfR was proposed (5). In a meta analysis (13) this parameter was found to improve the diagnosis of iron deficiency anaemia in the presence of coexisting chronic disease, but the parameter was found to suffer from low specificity, especially in cases with increased erythropoiesis. Consistent with these findings the sTfR did not add any additional information in our study population, especially without comorbidities. The same was true for the sTfRI which is derived by logarithmic transformation of sTfR to the base of ferritin (32).

As alternative tests we evaluated serum iron, transferrin and transferrin saturation, since these tests are commonly used to assess ID. We found these alternatives to be of little use. For example serum iron exhibits diurnal variations and may reach reference values after ingestion of red meat by iron depleted subjects. In our study serum iron had a poor diagnostic impact (AUC_ROC_ of 0.56), which is even lower than the reported (AUC_ROC_ of 0.7) (32). Transferrin saturation had a similarly poor AUC_ROC_ of 0.67. Transferrin had some diagnostic value as the AUCROC was found to be 0.8, a finding consistent with earlier studies in patients with ID anaemia (32). Nevertheless none of the above assays offers any advantage over determination of ferritin alone and should no longer be used for diagnosis (5).

The determination of microcytic and hypochromic erythrocyte percentage and reticulocyte haemoglobin content (CHr) have become popular parameters in hospitals with the use of an H1-H3 Advia 120 haematology analyser. Despite the limited diagnostic values (AUC_ROC_ around 0.66) in our as well as in other studies (43) the wide availability of the parameter might well help to identify ID stage II (ID with absent anaemia) in patients where ID was not suspected and therefore by consequence where ferritin levels would not have been determined.

In conclusion we found a very high prevalence of ID in a randomly chosen population of healthy hospital employees in Switzerland. We used ferritin levels as the non invasive gold standard in our population and realised that ID was reliably diagnosed by the mere measurement of serum ferritin, without the need for any further laboratory parameters. The addition of an array of laboratory parameters such as serum iron transferrin, transferrin saturation, or soluble transferrin receptor did not add any further information. The easiest and cheapest way to differentiate between latent (ID stage I) and manifest ID (stage II or III) is achieved by the quantification of the erythrocyte parameters.
